# Supercritical Fluids: An Innovative Strategy for Drug Development

**DOI:** 10.3390/bioengineering11080788

**Published:** 2024-08-04

**Authors:** Hui Liu, Xiaoliu Liang, Yisheng Peng, Gang Liu, Hongwei Cheng

**Affiliations:** 1 State Key Laboratory of Vaccine for Infectious Diseases, Xiang An Biomedicine Laboratory, National Innovation Platform for Industry-Education Integration in Vaccine Research, State Key Laboratory of Molecular Vaccinology and Molecular Diagnostics, School of Public Health, Xiamen University, Xiamen 361102, China; liuhui980220@163.com (H.L.); liangxiaoliu@stu.xmu.edu.cn (X.L.); 32620220156289@stu.xmu.edu.cn (Y.P.); 2 Zhuhai UM Science & Technology Research Institute, University of Macau, Macau SAR 999078, China

**Keywords:** supercritical fluid technology, nanomedicine, drug delivery, bioavailability, clinical application

## Abstract

Nanotechnology plays a pivotal role in the biomedical field, especially in the synthesis and regulation of drug particle size. Reducing drug particles to the micron or nanometer scale can enhance bioavailability. Supercritical fluid technology, as a green drug development strategy, is expected to resolve the challenges of thermal degradation, uneven particle size, and organic solvent residue faced by traditional methods such as milling and crystallization. This paper provides an insight into the application of super-stable homogeneous intermix formulating technology (SHIFT) and super-table pure-nanomedicine formulation technology (SPFT) developed based on supercritical fluids for drug dispersion and micronization. These technologies significantly enhance the solubility and permeability of hydrophobic drugs by controlling the particle size and morphology, and the modified drugs show excellent therapeutic efficacy in the treatment of hepatocellular carcinoma, pathological scarring, and corneal neovascularization, and their performance and efficacy are highlighted when administered through multiple routes of administration. Overall, supercritical fluids have opened a green and efficient pathway for clinical drug development, which is expected to reduce side effects and enhance therapeutic efficacy.

## 1. Introduction

Particle formation technology occupies an important position in drug development, and particle size reduction can help to improve the bioavailability of drugs in the body and reduce the toxic side effects caused by repeated administration or high dose administration [1]. For difficult-to-solve drugs, improving the morphology or reducing the size of drug particles, is one of the effective ways to improve bioavailability [2,3]. In addition, the enhanced permeability and retention (EPR) effect of nanoparticles provides new ideas to solve the drug delivery challenges, when drug-loaded nanoparticles flow through tumor blood vessels in the circulatory system, they accumulate in the tumor’s interstitial fluid. Following transmembrane transport and specific binding, the nanoparticles are internalized into the tumor tissue, achieving the passive targeted drug delivery [4]. In order to adjust particle morphology and particle size, traditional processing methods include grinding, crushing, sublimation, and crystallization [5,6]. However, the morphology and particle size are difficult to be controlled by these methods in physical processing. Especially for oral medications, irregular drug shapes take longer to dissolve and are not conducive to drug absorption. In addition, conventional pellet manufacturing techniques have some limitations in that the high process temperatures employed can lead to thermal degradation of heat-sensitive active ingredients [7]. For example, the heat generated during grinding or spray drying may affect the processing and drying of thermosensitive drugs, pharmaceuticals, and proteins and their mixtures. Supercritical technology allows for efficient precipitation of anthocyanins in a polymer matrix, reducing thermal degradation and eliminating residual ethanol from the extract [8]. The introduction of organic solvents, on the other hand, limits the development of chemical processing, especially in the development of drugs for food and in vivo use, where many solvents are restricted. For example, as a long-term health supplement, trans-resveratrol should reduce the use of organic solvents and surfactants as additives to minimize side effects [9].

The emergence of supercritical fluid (SCF) technology has overcome these limitations and has become a new favorite in the field of drug development and processing due to its many advantages such as sustainability, environmental friendliness, cost-effectiveness, and non-reactivity [10,11,12]. Supercritical state refers to the state in which the temperature and pressure of the fluid are higher than the critical value, at which time the fluid has the dual characteristics of liquid and gas, with the density close to that of a liquid and the viscosity similar to that of a gas [12]. Since its diffusion coefficient is close to that of gas, it has good mobility and mass transfer characteristics, and can be formulated with different forms and size distributions or even polycrystalline particles [7,13]. Common SCFs include carbon dioxide, nitrogen, ethylene, trichloromethane, and so on. Among them, supercritical carbon dioxide is the most common SCF, with a low supercritical temperature, the critical point is 31.3 °C, and the pressure critical point is 7.38 mPa, which it is not easy to modify or degrade heat-sensitive substances when in contact with many heat-sensitive substances [14]. Supercritical carbon dioxide is easy to obtain and is a green and safe fluid, so it is widely used in the fields of compound separation, drug mixing, and particle preparation.

**Figure 1:**
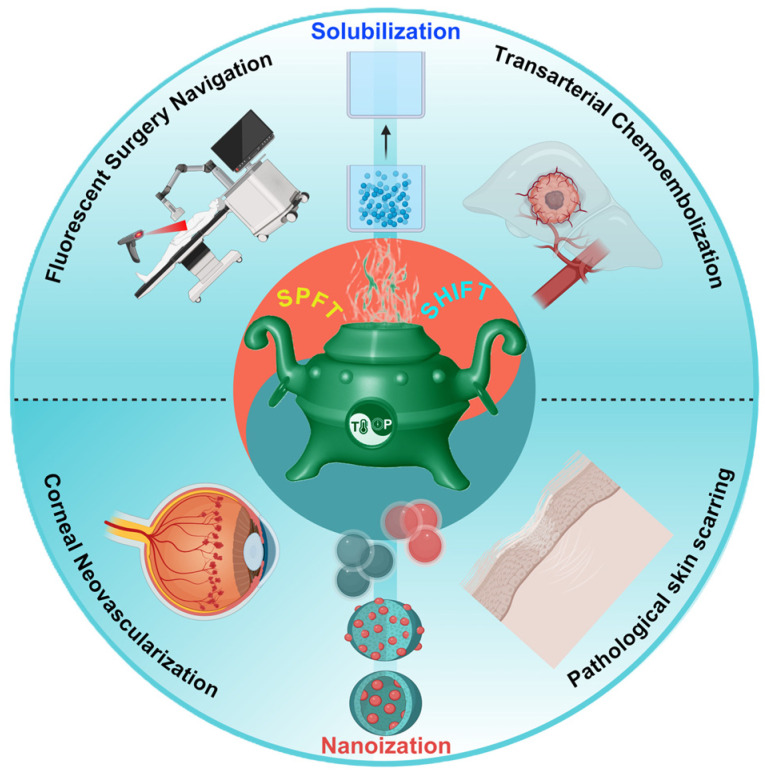
Schematic representation of a supercritical fluid technology for drug solubilization and nanonization. The red part represents the supercritical dispersion strategy and the blue part represents the supercritical nanosizing strategy.

Based on the advantages of using SCF in clinical drug preparation and the achieved results, this paper discusses the development of supercritical fluid-assisted drug dispersion and drug crystallization processes. It also exhibited the advantages of drug formulations prepared by using these processes and their performance in studies of diseases like hepatocellular carcinoma (HCC), hypertrophic scars, and corneal neovascularization, as well as in clinical applications such as surgical resection mediated by fluorescence navigation and chemoembolization (Figure 1). The paper provides examples and new insights for drug development and dosage form exploration.

## 2. Supercritical Procedures for Particle Formation and Dispersion

The dielectric constant of SCF relates to pressure changes. Altering the temperature and pressure of SCF causes changes in its density and solvent characteristics, controlling solute dissolution or precipitation. Due to these properties, SCF is widely used in areas like chemical separation, organic matter extraction, and powder preparation [14]. Recently, SCF precipitation technology is increasingly used in drug delivery for its advantages [15]. Supercritical carbon dioxide can be a processing solvent, reaction medium, or reactant in particle preparation. Various processes have been developed, mainly including rapid expansion process of supercritical solution (RESS) [16], supercritical anti-solvent (SAS) [17], and precipitation from gas saturated solution (PGSS) [18]. These processes are applied to pure drugs for micronization or particle morphology change, and in the preparation of eutectic formulations [19]. Additionally, supercritical fluid-assisted dispersion technology, non-toxic, and thermodynamically stable, is used for dispersing insoluble drugs.

The RESS method primarily employs the varying solvation ability of SCF with changes in ambient conditions like temperature and pressure. The solute for treatment dissolves in the SCF at specific temperature and pressure. It is then quickly sprayed through a special nozzle for decompression and expansion. As the density of the supercritical solvent changes, the solute in the jet becomes highly supersaturated and precipitates, generating numerous nuclei. Nuclei growth is accomplished in a short time, thereby preparing uniform fine particles. The SAS method is a process in which a SCF is used as a counter-solvent to dissolve the solid solute to be treated in a solvent, usually an organic solvent, and a SCF is selected as the counter-solvent, which is not soluble in the solute, but is miscible with the solvent. The solvent is ejected from the nozzle, and the anti-solvent phase contact and diffusion, solute solubility in the solvent decreases and rapid supersaturation precipitation, the formation of high-purity, uniform size distribution of particles. This method is suitable for substances that do not dissolve in SCF, the introduction of organic solvents extends the application of SCF in the preparation of polar particles, as a solvent of organic solvents can also be dissolved in SCF to be taken away, reducing the solvent residue. PGSS as the name implies SCF as a solute is dissolved in a liquid solution to form a saturated dissolution, and then quickly through the nozzle to expand and depressurize the solution of fluid from a supercritical state to a gaseous state. The solvent in the original solution vaporizes from the supercritical fluid that has changed into a gaseous state, with the solute precipitating out [20].

## 3. Supercritical Fluid-Assisted Drug Dispersion Technology

**Figure 2:**
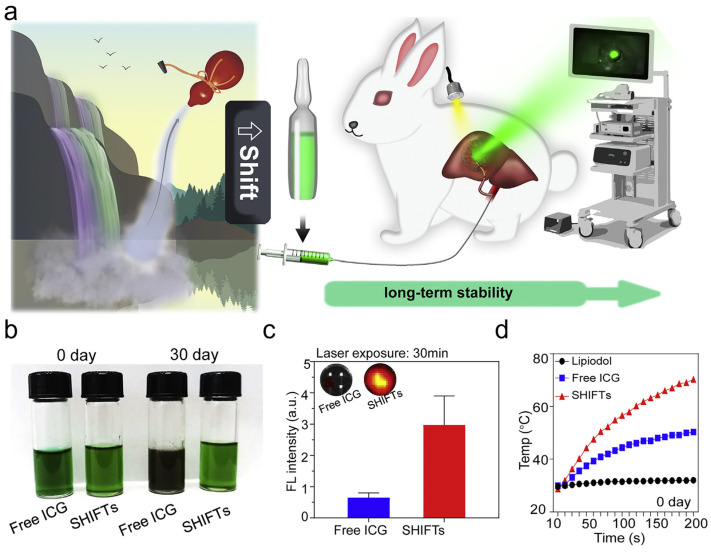
Drug dispersion assisted by super-stable homogeneous intermix formulating (SHIFT) technology. (**a**) The schematic illustration of SHIFT. (**b**) The photograph of free ICG and SHIFTs of newly prepared and stored after 30 days. (**c**) The fluorescence intensity of free ICG and SHIFTs. (**d**) The infrared thermal temperature of SHIFT, free ICG and lipiodol after laser irradiation. Reprinted with permission from Ref. [17]. With the development of radiotherapy technology and interventional medicine, transarterial radioembolization (TARE), as a branch of arterial embolization therapy, combines radioactive particles with embolizing agents to reach the focal site embolized by vascular interventions and produce radiation for therapeutic purposes [25,26]. Lipiodol as a labeling carrier combined with radionuclides for therapeutic drugs, such as 131I, 125I-lipiodol markers have been widely used in clinical treatment. The ^131^I-iodine oil retained in the hepatic artery for internal irradiation of tumors has higher therapeutic efficacy and lower probability of postoperative complications compared with oral sodium iodide (^131^I) solution [27,28]. SHIFT technology can replace the traditional mixing method of heating and stirring to improve the stability of radionuclides in iodine oil to achieve the effect of long-lasting internal radiation therapy, and the imaging ability of radionuclides can also help the specificity of the embolization site. The universal application of SHIFT technology allows the loading of multiple nuclide warheads for therapeutic or monitoring functions, showing excellent application prospects.

The effectiveness of a drug solution is closely related to the dispersion of drug particles in the solvent [21]. In the case of lipiodol, an oil-based embolic agent extensively employed in the treatment of HCC, the homogeneous dispersion of the chemotherapeutic drug was impossible to obtain by using conventional solubilization methods along with the addition of excipients [22]. Limited by the rapid separation of the hydrophilic drug from the hydrophobic oil phase, it is more difficult for drugs to be retained in the oil phase and exert long-lasting effects, especially for small molecule diagnostic probes that are desired to be retained for long periods of time intraoperatively and to guide surgical resection. Indocyanine green (ICG), a tracer approved by the U.S. Food and Drug Administration (FDA) for use in humans, can accumulate in tumor tissues, and is therefore widely used for the identification and navigation of tumor foci during HCC resection [23]. The crude emulsion prepared by pre-mixing ICG with iodine oil through a three-way valve has poor physical stability, and the precipitated ICG is metabolized and removed prior to the operation. The insufficient retention time of ICG will lead to weakening of the visualization time duration, which greatly diminishes the precision of the surgical operation for laparoscopic surgeries that lack haptic feedback [24]. In order to enhance the dispersion of hydrophilic small molecules in hydrophobic oil phases, an ultra-stable homogeneous formulation technique was developed using SCF technology [17], which was applied to avoid the introduction of organic solvents and to achieve complete dispersion of ICG in iodine oil, which is a good strategy for the insoluble drugs in biomedical applications (Figure 2a). The formed iodine oil-ICG-SHIFT (SHIFTs) formulations showed better stability and anti-burst ability compared with free ICG, and the homogeneous dispersion effectively changed the interactions between ICG molecules, reduced the aggregation effect, and possessed more stable photophysical properties (Figure 2b,c). The photothermal experiments also showed that SHIFTs maintained a more excellent photothermal conversion ability, proving the great potential of SHIFT technology in overcoming the low photothermal conversion efficiency of photothermal formulations (Figure 2d).

## 4. Supercritical Fluid-Assisted Drug Crystallization

The bioavailability of active compounds is affected by several factors, with drug solubility and permeability being the main factors hindering the poor utilization of most active compounds, and low drug accumulation at specific sites affecting therapeutic efficacy. In recent years, many drug particles have been processed to nano- or micron-size to increase the specific surface area in contact with the solution to facilitate drug solubilization, which has gained prominence in oral drug delivery and anti-cancer therapies [13,14,29]. An advanced micronization technique is the SCF antisolvent-based SPFT technique [30,31,32]. Especially for clinical drugs, with the assistance of SCFs, drug reassembly for micronization can be achieved without any additives. More significantly, supercritical carbon dioxide can also take away most of the organic solvents, further obtaining particles without solvent residues, which is something that both the drug makers and the drug users want to achieve, as it increases the safety of the drug in the body.

Currently, various active compounds [33,34,35,36], including small molecule fluorescent probes [32], chemotherapeutic agents [37], and antibiotics [38], have been prepared by micronization using SAS to obtain nanoparticles. These nanoparticles are smaller crystals with a more regular morphology, offering significant advantages in terms of improved solubility, enhanced bioavailability, optimized performance, and controlled production costs (Table 1).

**Figure 3:**
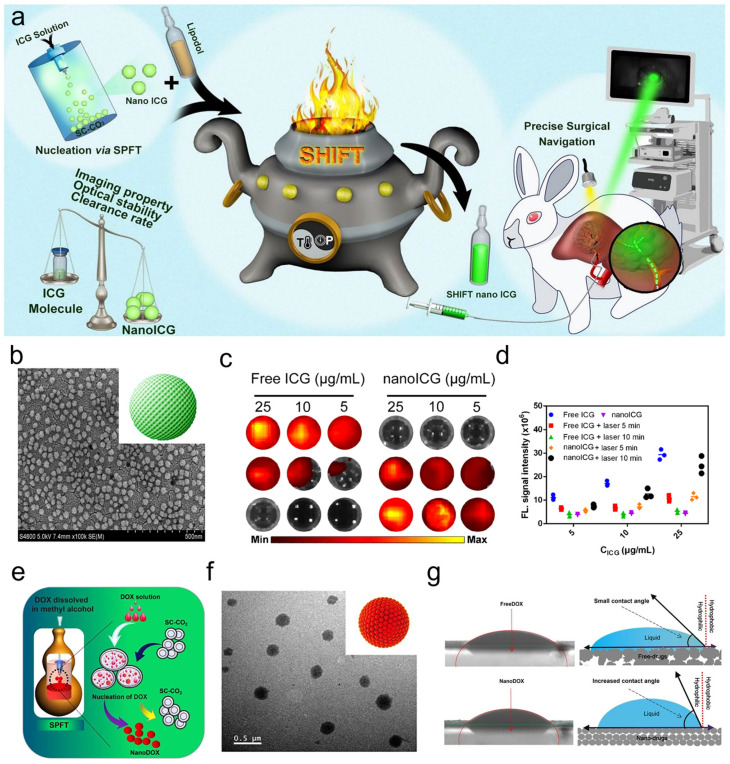
Drug crystallization by supercritical fluids. (**a**) Schematic illustration of SHIFT nanoICG preparation. (**b**) Dimensional characterization of nanoICG. (**c**,**d**) Anti-photobleaching experiments of free ICG and nanoICG, and the semi-quantitative analysis. Reprinted with permission from Ref. [32]. Copyright 2022 Springer Nature. (**e**) Preparation of nanoDOX via SPFT. (**f**) Dimensional characterization of nanoDOX. (**g**) The contact angle of free DOX and nanoDOX. The blue line simulates the horizontal plane that carries the bottom of the droplet [39].

Drug crystallization techniques with SCF allow the preparation of nanoparticles with uniform morphology. Nanosizing techniques have been applied to the preparation of probes to improve intermolecular interactions, and given that the ultra-stable homogeneous formulation technique demonstrated good performance in iodine oil-ICG mixtures, carrier-free ICG nanospheres (nano-ICG) were prepared using the SPFT technique (Figure 3a), and the modified nano-ICG had a particle size of 40.7 ± 4.5 nm, and demonstrated enhanced photoacoustic imaging capability and anti-bleaching ability, which is committed to a longer surgical visualization window (Figure 3b–d) [32]. In a weakly acidic environment, nano-ICG still maintains reliable fluorescence intensity, which facilitates the visualization and resection of tumor lesions. In addition, the increased surface area helps to reduce the intermolecular aggregation effect and better contact with water molecules. The thermal expansion capability of nano-ICG is enhanced, resulting in a higher photothermal conversion efficiency.

In addition to small-molecule fluorescent probes, drug crystallization technology with SCF holds even broader modification prospects for chemotherapeutic drug modification, which facilitates improved drug dispersion [42,43]. More attractively, nanosized drugs show better dispersion in aqueous solutions and can also be carried over into oil-based formulations, which is uniquely relevant for HCC treatment using iodine oil for drug delivery. While chemotherapeutic drugs are expected to have good solubility for systemic administration, the addition of hydrophilic excipients has hampered the application of drugs in HCC interventions, with poor dispersion of drugs in lipiodol leading to rapid release and triggering adverse effects beyond therapeutic purposes. Nanosizing doxorubicin (DOX) using SCF has been reported to improve its dispersion in lipiodol (Figure 3e) [31]. Compared to free DOX, which is large and non-uniformly aggregated, these carrier-free nano-doxorubicin (nano-DOX), have a well-defined spherical structure, smaller particle sizes (155 ± 29 nm) and exhibit higher contact angles in lipiodol (Figure 3f,g). These characteristics ensure good dispersion of nano-DOX in lipiodol, which helps to improve its retention time in lipiodol for a longer killing effect.

**Figure 4:**
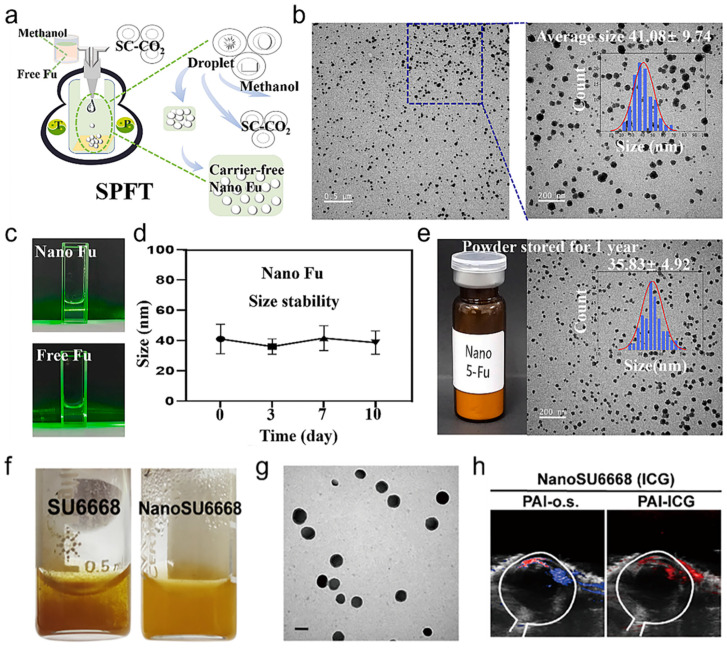
(**a**) A schematic diagram illustrating the synthesis of nano 5-Fu via SPFT. The arrows in the figure indicate the procedure for preparing Nano Fu using the SPFT technique. (**b**–**e**) Characterization of carrier-free nano 5-Fu. Reprinted with permission from Ref. [40]. Copyright 2023 Elsevier. (**f**) Schematic illustration of the preparation of nanoSU6668. (**g**) Characterization of SU6668 and nanoSU6668. (**h**) In vivo dual-PAI images of neovascularized eyes after topical administration of NanoSU6668. The white area is the approximate eye area, and both the red and blue areas are PA oxygen saturation signals, with blue indicating a low signal and red a high signal. Reprinted with permission from Ref. [41]. SPFT technique enhances the solubility of hydrophobic drugs in water by reducing the particle size [41]. SU6668, an anti-angiogenic oncology therapeutic drug, has a high degree of hydrophobicity that complicates the development of clinical formulations, and the addition of numerous excipients in the formulations poses many challenges in large-scale production. A SPFT strategy was used to synthesize carrier-free pure SU6668 nanoparticles (nano-SU6668), which exhibit uniform particle size (135 nm) with enhanced aqueous dispersibility, and thus exhibit excellent potential for improving drug delivery efficiency and prolonging in vivo circulation time (Figure 4f–h).

In particular, these carrier-free nanoparticles demonstrated long-lasting stability and exhibited higher drug dissolution rates. In another study, 5-Fu was nanomodified. After one year of storage, morphology and particle size like those of the initial formulation were still witnessed, which proved that the nanoparticles had good stability (Figure 4a–e) [44]. This indicated a high degree of physical and chemical stability, showcasing a good and stable potential for application. Therefore, it can be concluded that the drug crystallization technique using SCF offers advantages in both the morphology of the final drug product and the size of the particles that constitute it.

Similar to other supercritical-assisted drug micronization techniques, the SPFT technique has a wide range of applicability and can accommodate a wide variety of small particles achieved by selecting a suitable liquid solvent and varying the affinity between the solvent and the supercritical counter-solvent [7]. Commonly used solvents include ethanol, acetone, dimethyl sulfoxide, allowing micronization of active compounds in nano- or micrometer form. Mixtures of multiple solvents have also been attempted in some studies to dissolve the drug to reach saturation faster to precipitate the compound [20,42,43].

On the basis of single drug crystallization, SCF have also developed various processes for the production of polymer eutectic systems for a variety of biomedical applications, as compared to the traditional process samples of solid state milling and evaporative crystallization which may have disadvantages such as thermal degradation, organic solvent residues, unpredictable particle sizes [19,45]. SAS, as a strong eutectic crystallization procedure, allows to produce clean eutectic composites, which can be used for oral, intravenous administration, or topically, especially in the latter case, the combination of complexed drugs can confer additional therapeutic modalities or imaging capabilities. For example, given the enhanced solubility and photoacoustic imaging capabilities of nano-ICG, further co-preparation of ICG with SU6668 to obtain composite nanoparticles for corneal neovascularization can enable real-time monitoring of the drug’s tissue penetration and metabolism process, providing a possibility of visualizing the metabolic assessment procedure of the drug in vivo [41]. In addition, the supercritical co-crystallization strategy of p-toluenesulfonamide with the copolymer 4,4′-bipyridine can increase the dissolution rate by about 375-folds compared to the physical mixing approach [45]. However, the precipitation of composite polymers is not always successful. The precipitation of nanoparticles is a process that involves the rupture of the liquid jet and the nucleation of the particles. During this process, the distribution of various materials is not regular, and it remains to be investigated how to quantify the active substances. Effective precipitation is related to particle size and shape. If compounds tend to precipitate separately, they will produce their own drug single crystals. Therefore, supercritical fluid-based co-crystallization strategies, although clean and promising, still require screening of raw materials and improvement of operating parameters, including the liquid solvent, pressure, temperature, and drug concentration.

## 5. Clinical Applications of Supercritical Fluid Technology

### 5.1. Fluorescent Surgery Navigation

Fluorescent probes are widely used in bioimaging, and fluorescence imaging technology offers the advantages of real-time feedback, non-invasive and harmless, and relatively simple operation [31]. In order to meet the needs of vascular and vasculography as well as tumor monitoring, near-infrared probes with higher resolution and greater depth of penetration into biological matrices can also be selected for internal tissue visualization [46]. Among them, fluorescent surgical navigation gradually fills the gap between preoperative imaging confirmation and intraoperative tumor resection planning. ICG fluorescence-guided surgery helps to accurately identify tumor boundaries, facilitates precise resection of tumors, detects microscopic metastatic lesions, and reduces postoperative recurrences, demonstrating a great potential for clinical application [47]. To address the problem of occlusion of lipiodol to the blood vessel after embolization, which makes it difficult for fluorescein to enter, preoperative co-delivery of lipiodol mixed with ICG is a feasible method [48]. The high viscosity of lipiodol can effectively inhibit the aggregation of ICG molecules and improve the stability of fluorescence. However, embolization and resection of hepatocellular carcinoma are often not consecutive, and for patients who do not tolerate surgery, the tumor needs to be ischemically necrotic to a resectable size by interventional embolization, which usually requires a certain observation time. This requires good solubility and dispersion of ICG in lipiodol, which can be achieved by the SHIFT technology. The ICG-lipiodol formulation prepared by the SHIFT technology exhibits homogeneous dispersion, excellent stability, and specific deposition within the tumor [17,30,44,49]. The SHIFT-ICG formulation has excellent retention and photostability compared with the low enrichment of preoperative intravenously administered ICG at the tumor site due to vascular obstruction. It retained a highly luminous signal at the liver tumor site 14 days after embolization and was able to maintain a fluorescent signal under continuous irradiation for 5 h, implying a longer operational visualization bed. In human studies, the SHIFT-ICG-based formulation deposited and retained fluorescence capability at the tumor site after prolonged treatment with transcatheter arterial embolization (TAE) and was able to differentiate between tumor and normal liver tissue [44,49]. (BTM) According to further clinical studies (Clinical Registry No. ChiCTR2000035055), with the assistance of ICG fluorescence, patients receiving the ICG-iodine oil homogeneous formulation had clearer boundaries between tumors and normal tissues, and the formulation also assisted physicians in locating microsatellite foci not detected on preoperative imaging [50].

**Figure 5:**
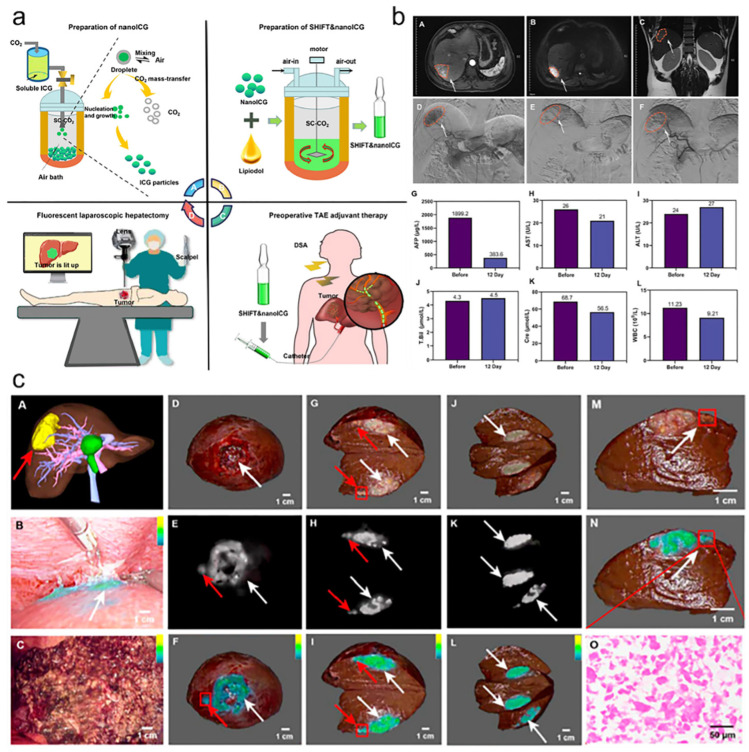
(**a**) Schematic illustration of SHIFT nanoICG preparation and fluorescence-guided precise hepatectomy. (**b**) Embolism and safety evaluation in clinical case of HCC. Red boxes and arrows are used to indicate the site of the tumor. (**c**) Surgical navigation effect of SHIFT nanoICG after long-lasting TAE-assisted therapy for varying degrees of lesions. White arrows are used to indicate tumor sites, red boxes and red arrows are used to indicate small tumor foci (A, preoperative three-dimensional reconstruction of hepatic resection; B, fluorescence imaging of the primary lesion; C, fluorescence imaging of the cut edge of the residual liver; D–F, whole resected tumor foci and fluorescence imaging; G–I, resected tumor foci and fluorescence imaging; J–L, layer-by-layer resected tumor foci and fluorescence imaging; M–N, microsatellite foci and fluorescence imaging; O, HE staining of microsatellite foci) [30].

The enhanced permeability and long retention properties of the nanoparticles are useful for clinical applications. Nano-ICG demonstrated excellent fluorescence imaging capabilities and resistance to photobleaching, which further led to the introduction of the nano-ICG-iodine oil homogeneous formulation (Figure 5a). It shows enhanced optical performance and long-lasting retention in liver in situ tumor models [32]. The combination of multiple preoperative imaging modalities, digital subtraction angiography (DSA) guidance and fluorescence precision surgery dedicated to resection of primary lesions and microscopic metastases yielded encouraging results. After clinical ethical approval, nano-ICG-iodine oil homogeneous preparation was placed intravenously in HCC patients and maintained a high signal-to-noise ratio during resection surgery two weeks later [30], effectively guiding the removal of deep tumor lesions and microsatellite foci (Clinical Registry Number: ChiCTR2200058803) (Figure 5b,c). Therefore, fluorescein-lipiodol homogeneous preparation prepared by SHIFT technology is an effective preparation for HCC transformation and precise resection, which may help to promote translational efforts in HCC treatment.

### 5.2. Transcatheter Arterial Chemoembolization

For patients who cannot tolerate surgical resection, lipiodol can be used as a carrier to deliver chemotherapeutic drugs. However, conventional lipiodol drug emulsions prepared manually through a three-way valve have poor physical stability, separating within 30 min, lack slow-release capability, and are susceptible to in vivo toxicity when large amounts of drug are released into the bloodstream. The production of lipophilic-based chemotherapeutic drugs is difficult and paradoxical because they are designed to be hydrophilic to meet the requirements for intravenous systemic administration. Therefore, there is an urgent need for a method to promote stable dispersion of chemotherapeutic drugs in lipiodol.

**Figure 6:**
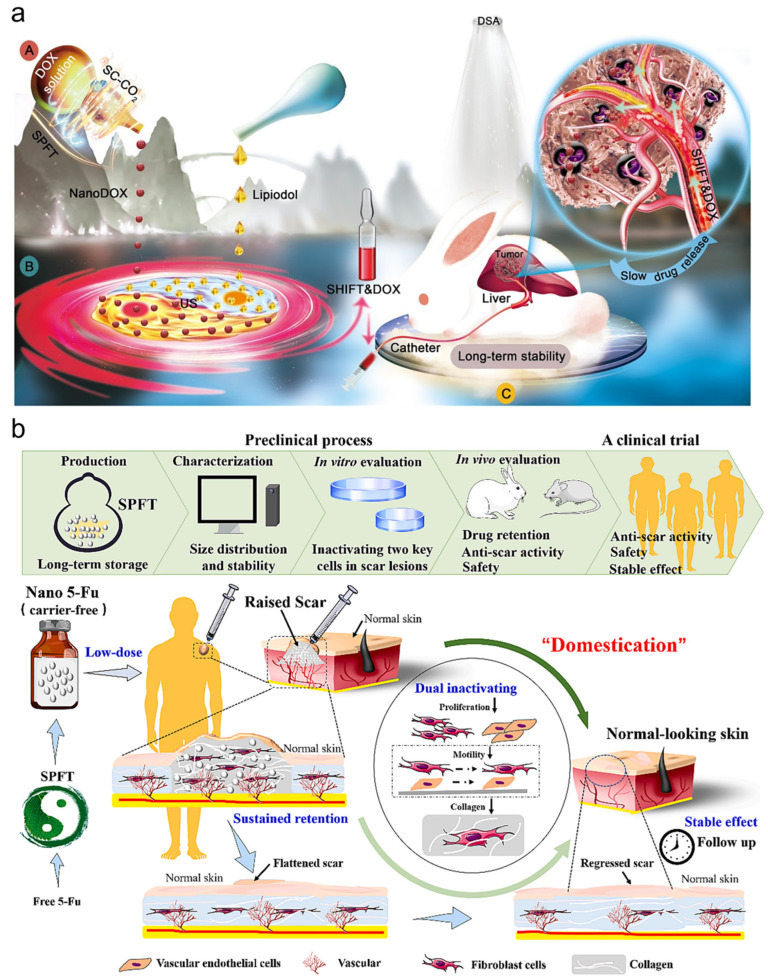
(**a**) Schematic illustration of SHIFT&DOX preparation and transarterial chemoembolization of HCC [39]. (**b**) The preclinical development stages of nano 5-Fu, including production, characterization, in vitro and in vivo evaluation, and subsequent clinical trials, are investigated. Reprinted with permission from Ref. [40].

Fortunately, the SHIFT technique was demonstrated in previous studies to be able to utilize the swelling and fast mass transfer properties of SCF to achieve the dispersion of ICG in lipiodol, providing a new direction for drug modification. The carrier-free nano-DOX prepared by the derived SPFT technique has a small particle size, high homogeneity, regular shape, and maintains a large contact angle in hydrophobic phase lipiodol, and these features ensure its stable dispersion in lipiodol. This formulation exhibited long drug retention time and slow drug release in ex vivo models, which is beneficial for transarterial chemoembolization (TACE) therapy (Figure 6a) [31]. Since the supercritical modification of the drug is a green physical process, the drug slowly precipitated from the lipiodol remains hydrophilic and can be rapidly absorbed by the tumor tissue. The SPFT technology is universally applicable to many other chemotherapeutic agents such as oxaliplatin, lenvatinib [51]. In addition, a recently approved clinical interventional study was conducted to identify the efficacy of the SHIFT oil iodide chemotherapeutic drug formulation in successful HCC transformation, fluorescence navigation, and safety, and to recommend a standard protocol for sequential precision surgery for the transformative treatment of intermediate and advanced unresectable hepatocellular carcinoma (Clinical Registry Number: ChiCTR2300070127).

### 5.3. Body Skin Scarring

Pathological scarring is a histopathological alteration of normal skin tissue caused by various traumatic injuries, which is widespread and poses a heavy medical and economic burden [52]. Fluorouracil (5-Fu), a commonly used chemotherapeutic agent, has also been used as an intralesional injection treatment for keloid scarring to inhibit fibroblast proliferation and collagen deposition [53]. However, free 5-Fu is rapidly cleared in soft tissues, and low-dose injections have been shown to only inhibit angiogenesis without reducing scar height, while high-dose injections often lead to side effects such as pain, ulceration, and hyperpigmentation [54,55]. The nano-5-Fu prepared by supercritical fluid-based SPFT strategy can effectively tame the scarring effect through rabbit ear scar model and human skin scarring for a long period of time (Figure 6b). The low dose of nano-5-Fu can transform the abnormal scar tissue into normal skin, which can help to alleviate the pain, itching and other disturbing side effects and improve the willingness to treat. A multicenter, randomized controlled clinical trial for this strategy is underway to obtain more clinical evidence on long-term efficacy, safety, and therapeutic mechanisms (Reg. No. ChiCTR2300069150) [44]. Transdermal drug delivery has become one of the key delivery modes in modern pharmacy, with the advantages of painless drug delivery, avoidance of hepatic first-pass metabolism, and good compliance. In addition to direct drug administration, some natural polymer carriers have shown potential in the field of topical drug delivery, and natural polymers such as PLA, PLGA, PAN, and cellulose, typically cellulose, have been used to construct these topical drug delivery systems [56,57,58]. However, the ability to penetrate therapeutic drugs is still limited, so active strategies such as microneedle delivery or electroporation have been developed to enhance transdermal drug penetration. The impregnation of microneedles with bioactive ingredients using SCF technology has advantages over traditional processing procedures, with more homogeneous particle size, higher matrix diffusivity, solubility, and less solvent residues [59]. However, there are some limitations to these methods, as the impregnation efficiency is low for drugs with high polarity, and the inflammation and skin breakage induced by microneedle use require some attention.

### 5.4. Corneal Neovascularization

The development of nano-delivery systems can facilitate drug penetration, and in addition to their application to the epidermal barrier, many ophthalmic diseases have been identified as targets for efficient drug delivery systems [60]. Corneal neovascularization arises from a variety of ophthalmic pathologies that disrupt the balance between angiogenic and anti-angiogenic factors, ultimately leading to vision loss and even blindness [61]. The application of traditional anti-VEGF therapeutic drugs has many shortcomings, such as repeated injections, low corneal penetration efficiency, and rapid drug clearance, which limit the efficacy of the drugs. SU6668, an anti-angiogenic drug after supercritical micronization, showed remarkable safety and therapeutic efficacy as eye drops [41]. This preparation process improves the water solubility of the hydrophobic drug, which is particularly suitable for the moist environment of the eye, helps to improve the drug delivery efficiency, and provides a new strategy for the development of corneal neovascularization therapeutic drugs. In addition, several nanocarriers can be employed to deliver VEGF antibodies to enhance the anti-angiogenic effects during CNV therapy. Liposomes are capable of encapsulating and loading hydrophilic drugs in the core region and hydrophobic drugs in the lipid bilayer [62]. The use of liposomes in ocular disease is closely related to lipid composition and surface charge, with positively charged liposomes facilitating drug uptake on negatively charged ocular surfaces [63]. However, the development of liposomes in the eye is limited by the shortcomings of hydrophilic drugs, such as low stability, low loading rate, and rapid release. SCF-treated liposomes are more efficiently encapsulated and have the potential to be loaded with multiple drugs compared to conventional liposome preparation methods [64,65]. Although these novel drug delivery strategies have shown promising properties in some aspects, they are currently limited to preclinical studies, such as animal models, and there is a lack of data from clinical human trials. In the future, clinical studies should be designed for multiple drug modification strategies, covering not only pharmacodynamic and toxicity studies, but also compliance studies. The prolonged drug release time and high penetration rate may trigger inflammation or even damage to the cornea. Secondly, excessive retention may also lead to stickiness and blurred vision, thus reducing patient compliance. Based on the derivation of supercritical sterilization technology, it may be possible to sterilize the drug and delivery vehicle at the same time as loading the drug.

## 6. Conclusions and Prospects

This paper offers a comprehensive examination of the homogeneous dispersion and drug crystallization processes founded on SCF technology for biomedical applications. SHIFT technology can achieve uniform dispersion of hydrophilic drugs in lipiodol and stable loading of small-molecule fluorescein in lipiodol, which effectively breaks through the clinical aspects of too-fast tissue removal, easy photobleaching, and a short window of surgical operation, and is committed to precise resection after TAE, effectively navigating the complexity of advanced hepatocellular carcinoma, and bringing the dawn of cure to advanced patients. This preparation can quickly and accurately establish the location and boundary of the primary tumor, achieving complete resection of tumor tissue in a short period of time (within 2 h) with minimal intraoperative blood loss (50 mL). The excised tumor specimen is surrounded by nano ICG fluorescence and maintains a good signal-to-noise ratio (mean = 1.79) with the tumor boundary. By using this process, some chemotherapy drugs with strong hydrophilicity can also be dispersed in lipiodol and extended to more chemotherapy drugs. The advancement of the SHIFT concept involves a deeper focus on additional drug loading platforms beyond oil-based formulations, such as iodine oil, with the goal of delivering a standardized, physical-level, and highly stable mixing solution. Moreover, this mixing approach holds promise in the development of templates for microsphere preparation, an area yet to be thoroughly explored by supercritical technology. In response to the solubility challenges of small molecule drugs, the integration of a micronized crystallization process based on SCF emerges as a versatile solution. By reducing particle size, it enhances solubility, thereby extending its application to various drugs. This modification strategy has demonstrated remarkable success in in vivo treatments like chemoembolization and in vitro drugs like skin scarring. It is anticipated to be employed in exploring the modification of more drugs and the construction of complex drug formulations. Clinical results from a study of nano 5-Fu in humans showed that nano 5-Fu at a low concentration (5 mg/mL) was more effective than free 5-Fu as a therapeutic regimen in reducing scar height, 5-Fu levels, and topical symptoms, with fewer topical complications and a higher degree of safety than free 5-Fu. These novel compounds address the existing issues of drug resistance, low permeability, and toxicity in the biomedical domain. It is evident that this green, safe, mass-producible, and carrier-free drug preparation process holds great potential as a promising strategy for clinical drug development. It not only fulfills the clinical requirements but also augments the therapeutic effects of drugs. Although SCF technology has shown significant advantages in the modification of drug particles, some further research is required to optimize the preparation parameters to obtain stable formulations. In the presence of multicomponent mixtures, covering multiple active ingredients and excipients added to enhance solubility, more in-depth investigations are required to obtain homogeneous particle sizes, specific structures, and reproducible products. It is expected that an exhaustive database on the final parameters of formulation modification can be constructed to meet the development needs of personalized drug delivery systems to achieve individually tailored drug concentration and release kinetics.

## Figures and Tables

**Table 1:** An overview of SPFT micronized active compounds.

Active Compound	Solvent	Pressure (MPa)	Temperature (°C)	Time (hours)	Size (nm)	Reference
ICG	EtOH/DCM	10	40	0.5	40.7 ± 4.5	[32]
DOX	MeOH	10	45	0.5	155 ± 29	[39]
5-Fu	MeOH	20	/	1	41.08 ± 9.74	[40]
SU6668	EtOH/DMSO	/	/	/	135	[41]

ICG: Indocyanine green; DOX: Doxorubicin; 5-Fu: Fluorouracil; SU6668: Orantinib; EtOH: Ethanol; DCM: Dichloromethane; MeOH: Methanol; DMSO: Dimethylsulfoxide.

## Data Availability

No new data were created or analyzed in this study. Data sharing is not applicable to this article.

## Abbreviations

SHIFT	Super-stable homogeneous intermix formulating technology
SPFT	Super-table pure-nanomedicine formulation technology
EPR effect	enhanced permeability and retention effect
SCF	Supercritical fluid
RESS	Rapid expansion process of supercritical solution
PGSS	Precipitation from gas saturated solution
SAS	Supercritical anti-solvent
HCC	Hepatocellular carcinoma
ICG	Indocyanine green
TARE	Transarterial radioembolization
DOX	Doxorubicin
TAE	Transcatheter arterial embolization
DSA	Digital subtraction angiography
TACE	Transarterial chemoembolization
5-Fu	Fluorouracil
SU6668	Orantinib

## References

[1] Campbell N., Deane C., Murphy P. (2017). The sounds of nanotechnology. Nat. Nanotechnol..

[2] Xu J., Song M., Fang Z., Zheng L., Huang X., Liu K. (2023). Applications and challenges of ultra-small particle size nanoparticles in tumor therapy. J. Control. Release.

[3] Anane-Adjei A.B., Jacobs E., Nash S.C., Askin S., Soundararajan R., Kyobula M., Booth J., Campbell A. (2022). Amorphous solid dispersions: Utilization and challenges in preclinical drug development within AstraZeneca. Int. J. Pharm..

[4] Fang J., Islam W., Maeda H. (2020). Exploiting the dynamics of the EPR effect and strategies to improve the therapeutic effects of nanomedicines by using EPR effect enhancers. Adv. Drug Deliv. Rev..

[5] Ha E.S., Park H., Lee S.K., Sim W.Y., Jeong J.S., Baek I.H., Kim M.S. (2020). Pure Trans-Resveratrol Nanoparticles Prepared by A Supercritical Antisolvent Process Using Alcohol and Dichloromethane Mixtures: Effect of Particle Size on Dissolution and Bioavailability in Rats. Antioxidants.

[6] Wu K., Li J., Wang W., Winstead D.A. (2009). Formation and characterization of solid dispersions of piroxicam and polyvinylpyrrolidone using spray drying and precipitation with compressed antisolvent. J. Pharm. Sci..

[7] Franco P., De Marco I. (2020). Supercritical Antisolvent Process for Pharmaceutical Applications: A Review. Processes.

[8] da Fonseca Machado A.P., Alves Rezende C., Alexandre Rodrigues R., Fernández Barbero G., de Tarso Vieira e Rosa P., Martínez J. (2018). Encapsulation of anthocyanin-rich extract from blackberry residues by spray-drying, freeze-drying and supercritical antisolvent. Powder Technol..

[9] Tran P., Park J.-S. (2021). Application of supercritical fluid technology for solid dispersion to enhance solubility and bioavailability of poorly water-soluble drugs. Int. J. Pharm..

[10] Zhang J., Liu M., Zeng Z. (2022). The antisolvent coprecipitation method for enhanced bioavailability of poorly water-soluble drugs. Int. J. Pharm..

[11] Knez Ž., Pantić M., Cör D., Novak Z., Knez Hrnčič M. (2019). Are supercritical fluids solvents for the future?. Chem. Eng. Process. Process Intensif..

[12] Badens E., Masmoudi Y., Mouahid A., Crampon C. (2018). Current situation and perspectives in drug formulation by using supercritical fluid technology. J. Supercrit. Fluids.

[13] Kang X., Mao L., Shi J., Liu Y., Zhai B., Xu J., Jiang Y., Lichtfouse E., Jin H., Guo L. (2024). Supercritical carbon dioxide systems for sustainable and efficient dissolution of solutes: A review. Environ. Chem. Lett..

[14] Martín A., Cocero M.J. (2008). Micronization processes with supercritical fluids: Fundamentals and mechanisms. Adv. Drug Deliv. Rev..

[15] Bagheri H., Hashemipour H., Ghader S. (2019). Population balance modeling: Application in nanoparticle formation through rapid expansion of supercritical solution. Comput. Part. Mech..

[16] Chen H., Cheng H., Dai Q., Cheng Y., Zhang Y., Li D., Sun Y., Mao J., Ren K., Chu C. (2020). A superstable homogeneous lipiodol-ICG formulation for locoregional hepatocellular carcinoma treatment. J. Control. Release.

[17] López-Iglesias C., Quílez C., Barros J., Velasco D., Alvarez-Lorenzo C., Jorcano J.L., Monteiro F.J., García-González C.A. (2020). Lidocaine-Loaded Solid Lipid Microparticles (SLMPs) Produced from Gas-Saturated Solutions for Wound Applications. Pharmaceutics.

[18] Dias J.L., Rebelatto E.A., Lanza M., Ferreira S.R.S. (2023). Production of quercetin-proline cocrystals by means of supercritical CO_2_ antisolvent. Adv. Powder Technol..

[19] Padrela L., Rodrigues M.A., Duarte A., Dias A.M.A., Braga M.E.M., de Sousa H.C. (2018). Supercritical carbon dioxide-based technologies for the production of drug nanoparticles/nanocrystals—A comprehensive review. Adv. Drug Deliv. Rev..

[20] Lin Y.-S., Tsay R.-Y. (2020). Drug Release from a Spherical Matrix: Theoretical Analysis for a Finite Dissolution Rate Affected by Geometric Shape of Dispersed Drugs. Pharmaceutics.

[21] Attia M.S., Elshahat A., Hamdy A., Fathi A.M., Emad-Eldin M., Ghazy F.-E.S., Chopra H., Ibrahim T.M. (2023). Soluplus^®^ as a solubilizing excipient for poorly water-soluble drugs: Recent advances in formulation strategies and pharmaceutical product features. J. Drug Deliv. Sci. Technol..

[22] Rossi G., Tarasconi A., Baiocchi G., De’ Angelis G.L., Gaiani F., Di Mario F., Catena F., Dalla Valle R. (2018). Fluorescence guided surgery in liver tumors: Applications and advantages. Acta Biomed..

[23] Goumard C., Komatsu S., Brustia R., Fartoux L., Soubrane O., Scatton O. (2017). Technical feasibility and safety of laparoscopic right hepatectomy for hepatocellular carcinoma following sequential TACE–PVE: A comparative study. Surg. Endosc..

[24] Schönherr J., Seifert P., Gühne F., Winkens T., Rauchfuß F., Settmacher U., Freesmeyer M., Drescher R. (2024). Transarterial Radioembolization (TARE) in Patients with Hepatocellular Carcinoma: A Comparison of Palliative with Bridging-to-Transplant Concepts. Cancers.

[25] Chen H., Teng M., Zhang H., Liang X., Cheng H., Liu G. (2022). Advanced radionuclides in diagnosis and therapy for hepatocellular carcinoma. Chin. Chem. Lett..

[26] Ahmadzadehfar H., Sabet A., Wilhelm K., Biersack H.J., Risse J. (2011). Iodine-131-Lipiodol therapy in hepatic tumours. Methods.

[27] Schwarz L., Bubenheim M., Gardin I., Huet E., Riachi G., Clavier E., Goria O., Vera P., Scotté M. (2016). Adjuvant I-131 Lipiodol After Resection or Radiofrequency Ablation for Hepatocellular Carcinoma. World J. Surg..

[28] Montes A., Wehner L., Pereyra C., Martínez de la Ossa E.J. (2016). Precipitation of submicron particles of rutin using supercritical antisolvent process. J. Supercrit. Fluids.

[29] He P., Xiong Y., Ye J., Chen B., Cheng H., Liu H., Zheng Y., Chu C., Mao J., Chen A. (2022). A clinical trial of super-stable homogeneous lipiodol-nanoICG formulation-guided precise fluorescent laparoscopic hepatocellular carcinoma resection. J. Nanobiotechnol..

[30] Li Y., Chen Q., Pan X., Lu W., Zhang J. (2022). Development and Challenge of Fluorescent Probes for Bioimaging Applications: From Visualization to Diagnosis. Top. Curr. Chem..

[31] Zhang Y., Cheng H., Chen H., Xu P., Ren E., Jiang Y., Li D., Gao X., Zheng Y., He P. (2022). A pure nanoICG-based homogeneous lipiodol formulation: Toward precise surgical navigation of primary liver cancer after long-term transcatheter arterial embolization. Eur. J. Nuclear Med. Mol. Imaging.

[32] He H., Huang Y., Zhang X., Ouyang Y., Pan P., Lan Y., Zhong Z., Ping L., Lu T., Chen Z. (2023). Supercritical fluid coating of flavonoids on excipients enhances drug release and antioxidant activity. Int. J. Pharm..

[33] Esfandiari N., Saadati Ardestani N., Alwi R.S., Rojas A., Garlapati C., Sajadian S.A. (2023). Solubility measurement of verapamil for the preparation of developed nanomedicines using supercritical fluid. Sci. Rep..

[34] Zhong Z., Lan Y., Chen J., Ping L., Li X., Wang Q., Zhuang X., Qiu Z., Yuan T., Guo Q. (2024). Optimizing Paclitaxel Oral Absorption and Bioavailability: TPGS Co-Coating via Supercritical Anti-Solvent Fluidized Bed Technology. Pharmaceuticals.

[35] Zhang F., Zhang A., Xie Y., Wen H., Kankala R.K., Huang J., Zhang A., Wang Q., Chen B., Dong H. (2023). Nanocarrier of Pin1 inhibitor based on supercritical fluid technology inhibits cancer metastasis by blocking multiple signaling pathways. Regen. Biomater..

[36] De Marco I. (2022). Supercritical Fluids and Nanoparticles in Cancer Therapy. Micromachines.

[37] Campardelli R., Trucillo P., Reverchon E. (2018). Supercritical assisted process for the efficient production of liposomes containing antibiotics for ocular delivery. J. CO2 Util..

[38] He P., Ren E., Chen B., Chen H., Cheng H., Gao X., Liang X., Liu H., Li J., Li B. (2022). A super-stable homogeneous Lipiodol-hydrophilic chemodrug formulation for treatment of hepatocellular carcinoma. Theranostics.

[39] He P., Yi S., Zhang J., Chu C., Peng X., Li C., Sun X., Zhang Y., Cheng H., Xiong X. (2023). Carrier-free 5-Fu nanoparticle-mediated domestication therapy for scar treatment: A preclinical and first-in-human study. Chem. Eng. J..

[40] Wu H., Ye J., Zhang M., Zhang L., Lin S., Li Q., Liu Y., Han Y., Huang C., Wu Y. (2024). A SU6668 pure nanoparticle-based eyedrops: Toward its high drug Accumulation and Long-time treatment for corneal neovascularization. J. Nanobiotechnol..

[41] Wang Z.-D., Peng H.-H., Guan Y.-X., Yao S.-J. (2022). Supercritical CO_2_ assisted micronization of curcumin-loaded oil-in-water emulsion promising in colon targeted delivery. J. CO2 Util..

[42] Amani M., Saadati Ardestani N., Majd N.Y. (2021). Utilization of supercritical CO_2_ gas antisolvent (GAS) for production of Capecitabine nanoparticles as anti-cancer drug: Analysis and optimization of the process conditions. J. CO2 Util..

[43] Xiong Y., He P., Zhang Y., Chen H., Peng Y., He P., Tian J., Cheng H., Liu G., Li J. (2023). Superstable homogeneous lipiodol-ICG formulation: Initial feasibility and first-in-human clinical application for ruptured hepatocellular carcinoma. Regen. Biomater..

[44] Khudaida S.H., Yen Y.-T., Su C.-S. (2024). Cocrystal screening of anticancer drug p-toluenesulfonamide and preparation by supercritical antisolvent process. J. Supercrit. Fluids.

[45] Li H., Kim Y., Jung H., Hyun J.Y., Shin I. (2022). Near-infrared (NIR) fluorescence-emitting small organic molecules for cancer imaging and therapy. Chem. Soc. Rev..

[46] Li W.-F., Al-Taher M., Yu C.-Y., Liu Y.-W., Liu Y.-Y., Marescaux J., Cheng Y.-F., Diana M., Wang C.-C. (2021). Super-Selective Intra-Arterial Indocyanine Green Administration for Near-Infrared Fluorescence-Based Positive Staining of Hepatic Segmentation: A Feasibility Study. Surg. Innov..

[47] Shi X., Xu D., Cheng H., Chu C., Liu G. (2023). Recent Advances in Interventional Fluorescence Imaging: Toward the Precise Visualization of Transarterial Mini-Invasive Delivery Systems. Acc. Mater. Res..

[48] He P., Xiong Y., Luo B., Liu J., Zhang Y., Xiong Y., Su S., Fang C., Peng Y., Cheng H. (2023). An exploratory human study of superstable homogeneous lipiodol-indocyanine green formulation for precise surgical navigation in liver cancer. Bioeng. Transl. Med..

[49] Peng Y., Cheng H., Liu H., Zhang Y., Liu G. (2023). Super-stable homogeneous embolic agents advance the treatment of hepatocellular carcinoma. iRADIOLOGY.

[50] Cheng H., Yang X., Liu G. (2020). Superstable homogeneous iodinated formulation technology: Revolutionizing transcatheter arterial chemoembolization. Sci. Bull..

[51] Vorstandlechner V., Laggner M., Copic D., Klas K., Direder M., Chen Y., Golabi B., Haslik W., Radtke C., Tschachler E. (2021). The serine proteases dipeptidyl-peptidase 4 and urokinase are key molecules in human and mouse scar formation. Nat. Commun..

[52] Searle T., Al-Niaimi F., Ali F.R. (2021). 5-Fluorouracil in Dermatology: The Diverse Uses Beyond Malignant and Premalignant Skin Disease. Dermatol. Surg..

[53] Liu W., Wu X., Gao Z., Xia L., Téot L., Mustoe T.A., Middelkoop E., Gauglitz G.G. (2020). Minimally Invasive Technologies for Treatment of HTS and Keloids: Low-Dose 5-Fluorouracil. Textbook on Scar Management: State of the Art Management and Emerging Technologies.

[54] Liang Y., Ding A., Lyu D., Wang D., Zhou R. (2023). Comparison of Ultrasound-Assisted Low-Dose Versus Medium-Dose 5-Fluorouracil and Triamcinolone Acetonide in the Treatment of Hypertrophic Scar. Dermatol. Ther..

[55] Khalid R., Mahmood S., Mohamed Sofian Z., Hilles A.R., Hashim N.M., Ge Y. (2023). Microneedles and Their Application in Transdermal Delivery of Antihypertensive Drugs-A Review. Pharmaceutics.

[56] Malek-Khatabi A., Sadat Razavi M., Abdollahi A., Rahimzadeghan M., Moammeri F., Sheikhi M., Tavakoli M., Rad-Malekshahi M., Faraji Rad Z. (2023). Recent progress in PLGA-based microneedle-mediated transdermal drug and vaccine delivery. Biomater. Sci..

[57] Sabbagh F., Kim B.S. (2022). Recent advances in polymeric transdermal drug delivery systems. J. Control. Release.

[58] Kankala R.K., Zhang Y.S., Wang S.B., Lee C.H., Chen A.Z. (2017). Supercritical Fluid Technology: An Emphasis on Drug Delivery and Related Biomedical Applications. Adv. Healthc. Mater..

[59] Gholizadeh S., Wang Z., Chen X., Dana R., Annabi N. (2021). Advanced nanodelivery platforms for topical ophthalmic drug delivery. Drug Discovery Today.

[60] Nicholas M.P., Mysore N. (2021). Corneal neovascularization. Exp. Eye Res..

[61] Onugwu A.L., Nwagwu C.S., Onugwu O.S., Echezona A.C., Agbo C.P., Ihim S.A., Emeh P., Nnamani P.O., Attama A.A., Khutoryanskiy V.V. (2023). Nanotechnology based drug delivery systems for the treatment of anterior segment eye diseases. J. Control. Release.

[62] Sheikholeslami B., Lam N.W., Dua K., Haghi M. (2022). Exploring the impact of physicochemical properties of liposomal formulations on their in vivo fate. Life Sci..

[63] Pasquali I., Bettini R. (2008). Are pharmaceutics really going supercritical?. Int. J. Pharm..

[64] Gaikwad S.S., Pathare S.R., More M.A., Waykhinde N.A., Laddha U.D., Salunkhe K.S., Kshirsagar S.J., Patil S.S., Ramteke K.H. (2023). Dry Powder Inhaler with the technical and practical obstacles, and forthcoming platform strategies. J. Control. Release.

[65] Jash A., Krueger A., Rizvi S.S.H. (2022). Venturi-based rapid expansion of supercritical solution (Vent-RESS): Synthesis of liposomes for pH-triggered delivery of hydrophilic and lipophilic bioactives. Green Chem..

